# Arthritis diagnosis and symptoms are positively associated with specific physical job exposures in lower- and middle-income countries: cross-sectional results from the World Health Organization’s Study on global AGEing and adult health (SAGE)

**DOI:** 10.1186/s12889-018-5631-2

**Published:** 2018-06-08

**Authors:** Sharon L. Brennan-Olsen, Svetlana Solovieva, Eira Viikari-Juntura, Ilana N. Ackerman, Steven J. Bowe, Paul Kowal, Nirmala Naidoo, Somnath Chatterji, Anita E. Wluka, Michelle T. Leech, Richard S. Page, Kerrie M. Sanders, Fernando Gomez, Gustavo Duque, Darci Green, Mohammadreza Mohebbi

**Affiliations:** 1 Australian Institute for Musculoskeletal Science (AIMSS), The University of Melbourne and Western Health, Level 3, WHCRE Building, C/- Sunshine Hospital, 176 Furlong Road, St Albans, Melbourne, VIC 3021 Australia; 2 Department of Medicine-Western Health, St Albans, Australia; 3 Finnish Institute of Occupational Health, Helsinki, Finland; 4 Department of Epidemiology and Preventive Medicine, Monash University, Melbourne, Australia; 5 Deakin University, Geelong, Australia; 6 Department of Health Statistics and Information Systems, World Health Organization, Geneva, Switzerland; 7 Research Institute for Health Sciences, Chiang Mai University, Chiang Mai, Thailand; 8 Monash University, Melbourne, Australia; 9 Barwon Centre for Orthopaedic Research and Education, Barwon Health, Geelong, Australia; 10 Health Faculty, University of Caldas, Manizales, Colombia

**Keywords:** Arthritis, Lower- and middle-income countries, Obesity, Occupation, Social factors

## Abstract

**Background:**

In higher income countries, work-related squatting and heavy lifting have been associated with increased arthritis risk. Here, we address the paucity of data regarding associations between arthritis and work-related physical stressors in lower- and middle-income countries.

**Methods:**

Data were extracted from the Study on global AGEing and adult health (SAGE) Wave 1 (2007–10) for adults (aged ≥50 years) from Ghana, India, Russia and South Africa for whom detailed occupation data was available (*n* = 21,389; 49.2% women). Arthritis cases were identified using a symptom-defined algorithm (current) and self-reported doctor-diagnosis (lifetime). A sex-specific Job Exposure Matrix was used to classify work-related stressors: heavy physical work, kneeling/squatting, heavy lifting, arm elevation and awkward trunk posture. Using the International Standard Classification of Occupations, we linked SAGE and the Job Exposure Matrix. Logistic regression was used to investigate associations between arthritis and work-related stressors, adjusting for age (10 year age groupings), potential socioeconomic-related confounders, and body mass index. Excess exposure risk due to two-way interactions with other risk factors were explored.

**Results:**

Doctor-diagnosed arthritis was associated with heavy physical work (adjusted odds ratios [OR] 1.12, 95%CI 1.01–1.23), awkward trunk posture (adjusted OR 1.23, 95%CI 1.12–1.36), kneeling or squatting (adjusted OR 1.25, 95%CI 1.12–1.38), and arm elevation (adjusted OR 1.66, 95%CI 1.37–2.00). Symptom-based arthritis was associated with kneeling or squatting (adjusted OR 1.27, 95%CI 1.08–1.50), heavy lifting (adjusted OR 1.33, 95%CI 1.11–1.58), and arm elevation (adjusted OR 2.16, 95%CI 1.63–2.86). Two-way interactions suggested excess arthritis risk existed for higher body mass index, and higher income or education.

**Conclusions:**

Minimization of occupational health risk factors is common practice in higher income countries: attention should now be directed toward reducing work-related arthritis burden in lower- and middle-income countries.

**Electronic supplementary material:**

The online version of this article (10.1186/s12889-018-5631-2) contains supplementary material, which is available to authorized users.

## Background

In higher income countries, a greater prevalence of knee osteoarthritis has been associated with work-related stressors such as kneeling or squatting, heavy lifting, and climbing [1–3], with similar findings for hip osteoarthritis [4, 5]. In contrast, very little is known about the relationship between physical work-related stressors and arthritis in lower- and middle-income countries (LMICs). This is despite occupational risk factors associated with osteoarthritis, such as repetitive trauma, knee bending or lifting heavy weights, being more prevalent in occupational groups such as farmers and unskilled workers [6–8], and therefore may be more often experienced by workers in LMICs. There appears an inextricable link between occupation and education in higher income countries [9–11], which may plausibly exist in LMICs with clear consequences for arthritis. For instance, poverty and lower educational attainment may predispose populations to manual labour tasks. Indeed, we have reported that higher arthritis prevalence was associated with lower educational attainment in over 44,000 residents from six LMICs enrolled in the World Health Organization (WHO) Study on global AGEing and adult health (SAGE) [12]. For those in LMICs, high arthritis prevalence may also worsen poverty by impacting on a person’s ability to work and fulfil community roles. Taken together, the potential bi-directional relationship between poverty and arthritis presents a concerning situation for populations of LMICs.

The social gradient of obesity in higher income countries is well-documented [13], however, the obesity epidemic is now a global concern, with the sharpest rise in prevalence observed in LMICs [13, 14]. The WHO reports that obesity paradoxically coexists with undernutrition in lower-income countries [14]. Given that obesity is a strong risk factor for the development of knee and hand osteoarthritis, likely through metabolic mechanisms [15], it is also important to consider the relationship between overweight and obesity and arthritis, particularly osteoarthritis, in LMICs to inform arthritis prevention efforts. Much of the available data regarding obesity and arthritis come from higher income countries (or from studies with high representation from Caucasian populations), therefore, it is imperative that this knowledge gap be addressed for LMICs. In this context, it is important to understand whether specific physical work-related stressors are more likely associated with higher likelihood of arthritis diagnosis and worse symptomatology, and also if obesity, and social determinants, play a role in these associations.

The Job Exposure Matrix (JEM) provides an internationally comparable framework for evaluating major work-related physical stressors (encompassing heavy physical work, kneeling or squatting, heavy lifting, arm elevation, and awkward trunk exposure). Linking the JEM to population data from the SAGE cohort, this study aimed to investigate the association between specific physical job stressors and arthritis diagnosis and symptoms in LMICs, and to explore the role of obesity and social determinants.

## Methods

### Study population and design

SAGE Wave 1 (2007–10) is a cross-sectional study involving nationally representative samples of persons aged ≥50 years and a smaller sample of adults aged 18–49 years: Wave 1 includes a total of 44,747 adults aged ≥18 years from China, Ghana, India, Mexico, Russian Federation and South Africa [16, 17]. The World Health Organization’s Ethical Review Board provided ethics approvals. Written, informed consent was obtained from all participants.

This study adheres to the STROBE reporting guidelines [18].

### Arthritis status: Doctor-diagnosed and symptom-based

Consistent with arthritis prevalence analyses for this cohort [12], doctor-diagnosed arthritis (lifetime) was based on participant responses to the question; *“Have you ever been diagnosed with/told by a health care professional you have arthritis (a disease of the joints; or by other names rheumatism or osteoarthritis)?”* As a secondary endpoint, a symptom-based determination of arthritis (current: yes/no) was also employed, using an algorithm developed by the WHO SAGE study team [16], to identify a pattern of symptoms that are indicative of having osteoarthritis, rather than symptoms most likely to indicate inflammatory arthritis. This algorithm is presented in Online Additional file 1: Table S1.

### Physical work-related stressors

The main occupation of each participant during the previous 12 months was self-reported to the SAGE field staff of each country. The International Standard Classification of Occupation (ISCO-88) provides a coding system for classifying occupations according to the dimensions of skill level and skill specialisation [19–21]. Skill level is a function of the range and complexity of the tasks involved. Skill specialisation reflects the type of knowledge applied, tools and equipment used, materials worked on (or with), and the nature of the goods and services produced [19–21]. Based on skill levels, the ISCO-88 [19–21] delineates the top aggregation level by categorising occupations into ten major (hierarchical) groups of 1 = legislators, senior officials and managers; 2 = professionals; 3 = technicians and associate professionals; 4 = clerks; 5 = service workers and shop and market sales workers; 6 = skilled agricultural and fishery workers; 7 = craft and related trade workers; 8 = plant and machine operators and assemblers; 9 = elementary occupations; 10 = armed forces. Further subdivision of these occupations (using a 4–6 digit code, rather than the single digit code described above that indicates the top aggregation level only) results in 390 unit groups based on skill-specialisation, representing the most detailed level of the ISCO-88 structure and incorporating thousands of detailed occupation descriptions [21]. At this level of delineation, specific occupational-related exposures are defined. Notably, 4–6 digit occupational data were collected for all countries with the exception of China and Mexico. Due to this reason, China and Mexico were excluded from this study, and from the initial 44,747 participants in the SAGE Wave 1, we were left with 21,514 individuals (49.2% female) from Ghana, India, Russian Federation and South Africa for inclusion in our current analyses.

### Job exposure matrix (JEM)

The ISCO-88 code was also used to link SAGE participants with the JEM (physical exposure matrix only) developed by Solovieva et al. [21, 22]. For a given job or occupation title, exposure level was assigned based on the group-specific average of exposure. Performance of the JEM was evaluated in terms of accuracy, sensitivity, specificity and predictive ability, and scored relatively high [21]. The JEM identifies five work-related exposures of (i) heavy physical work, (ii) kneeling or squatting, (iii) heavy lifting, (iv) arm elevation, and (v) awkward trunk posture.

### Body mass index

Recorded weights and heights of participants were used to calculate BMI (kg/m^2^), which were then categorised as underweight (< 18.5 kg/m^2^), normal range (18.5–24.99 kg/m^2^), overweight (25–29.99 kg/m^2^), Class 1 obese (≥30 kg/m^2^), and Class 2 obese [14, 23].

### Social determinants

Household income was self-reported and categorised into quintiles for analyses, whereby quintile 1 represented the lowest income and quintile 5 represented the highest. Participants were asked if they had ever been to school; for those that indicated ‘yes’, they were also asked to identify the highest level of education completed. Education was categorised as (i) ‘no formal schooling’, (ii) some primary school education but primary school not completed, (iii) primary school completed, (iv) secondary school or high school (or equivalent) completed, or (v) tertiary education completed, including college, pre-university, university, or post-graduate degree. Education levels were mapped to an international standard [24]. Self-reported marital status was categorised for analyses into three groups of: (i) never married, (ii) currently married or cohabiting, and (iii) separated/divorced or widowed. Region was defined as urban versus rural.

### Statistical analyses

We used multivariable logistic regression models to investigate associations between each of the physical work-related stressors measured in the JEM and arthritis, measured by a doctor-diagnosis or symptoms; adjustments were made for age (10-year age groupings) and sex, with further adjustment for categories of BMI (underweight and normal BMI were combined due to small cell sizes), and simultaneous adjustments for educational attainment (none, some primary, primary completed, secondary completed, tertiary completed), household income level (quintiles), marital status (never married, married/cohabiting, separated/divorced/widowed), region (urban vs. rural), and country. In multivariable models, for each variable, with the exception of country, the categorical group with the lowest risk of arthritis was held as referent group (male; aged 30–39 years; BMI < 24.99; highest income quintile; highest level of educational attainment; never married; urban resident). All two-way interactions for significant risk factors/covariates with the physical work-related stressors were investigated through multivariate logistic regression models: whereby BMI, income and education were treated as ordinal variables, with increasing values relating to higher levels of these variables. The two-way interaction models contained main effects for all significant risk factors/covariates from step 1, and all two-way interactions of model factors with all of the physical work-related stressors that were measured. A backward elimination variable selection method (*p*-value entry = 0.1, and *p*-value exit = 0.05) was then implemented to examine the interaction effects.

## Results

Table 1 presents the characteristics of the study population (*n* = 21,389; 49.2% female), stratified by country. For each country, with the exception of Ghana where awkward trunk posture was most common (72.6%), heavy physical work was the most common physical job stressor (range: 24.4% in Russian Federation to 61.3% in India). The greatest proportion of participants from Ghana and India had normal BMI (61.1 and 53.9%, respectively), whilst in Russian Federation and South Africa the greatest proportion of individuals had a BMI in the overweight or obese categories, respectively (40.3% Russian Federation and 38.7% South Africa); 32.8% of participants from India had a BMI < 25 kg/m^2^. For quintiles of household income levels, the greatest difference between the lowest compared to the highest income was in Russian Federation where the proportions were 14.9% vs. 27.7%. For each country, participants were more likely to be married or cohabiting compared to single or separated, divorced or widowed. Half of the participants from Ghana, ~ 40% of participants from India and South Africa, and 1.5% from the Russian Federation had no formal schooling. Many of the participants from Ghana and India resided in rural areas (62 and 69%, respectively), compared to the minority of participants from Russian Federation and South Africa (17 and 28%, respectively).

**Table 1:** Characteristics of the study population (*n* = 21,389), stratified by country

Characteristics	Ghana (*n* = 4140)	India (*n* = 9210)	Russian Federation (*n* = 5523)	South Africa (*n* = 2516)
Exposure to the physical exposures measured in the Job Exposure Matrix				
Heavy physical work	2955 (71.4%)	5650 (61.3%)	1345 (24.3%)	1136 (45.1%)
Kneeling or squatting	1278 (30.9%)	1440 (15.6%)	606 (11.0%)	783 (31.1%)
Heavy lifting	1762 (42.5%)	4077 (44.3%)	536 (9.7%)	329 (13.1%)
Arm elevation	148 (3.6%)	216 (2.3%)	354 (6.4%)	97 (3.9%)
Awkward trunk posture	3004 (72.5%)	2732 (29.7%)	698 (12.6%)	310 (12.3%)
Sex				
Male	2435 (58.8%)	5528 (60.0%)	1906 (34.5%)	998 (39.7%)
Female	1706 (41.2%)	3682 (40.0%)	3617 (65.5%)	1518 (60.3%)
Age groups (years)				
30–39	84 (2.0%)	786 (8.5%)	246 (4.4%)	20 (0.8%)
40–49	246 (5.9%)	1295 (14.1%)	242 (4.4%)	113 (4.5%)
50–59	269 (6.5%)	1028 (11.2%)	209 (3.8%)	55 (2.2%)
60–69	1340 (32.4%)	2817 (30.6%)	2010 (36.4%)	1193 (47.4%)
70–79	1105 (26.7%)	2133 (23.2%)	1277 (23.1%)	663 (26.3%)
≥80	1096 (26.5%)	1151 (12.5%)	1539 (27.9%)	472 (18.8%)
Body mass index (BMI)				
< 25 (kg/m^2^)	576 (14.2%)	2944 (32.8%)	65 (1.3%)	96 (3.9%)
25–29.99 (kg/m^2^)	2472 (61.1%)	4832 (53.9%)	1366 (26.7%)	603 (24.8%)
30–34.99 (kg/m^2^)	631 (15.6%)	1008 (11.2%)	2056 (40.2%)	807 (33.1%)
≥35 (kg/m^2^)	366 (9.0%)	183 (2.0%)	1631 (31.9%)	929 (38.1%)
Household income quintiles				
Quintile 1 (lowest income)	866 (20.9%)	1662 (18.1%)	819 (14.9%)	423 (16.8%)
Quintile 2	816 (19.7%)	1616 (17.6%)	980 (17.8%)	620 (24.7%)
Quintile 3	737 (17.8%)	1804 (19.6%)	949 (17.2%)	443 (17.6%)
Quintile 4	821 (19.8%)	2037 (22.2%)	1232 (22.4%)	411 (16.3%)
Quintile 5 (highest income)	897 (21.7%)	2061 (22.4%)	1530 (27.8%)	616 (24.5%)
Educational attainment				
No schooling	2065 (50.1%)	3678 (39.9%)	32 (0.6%)	457 (20.9%)
Some primary school	447 (10.8%)	964 (10.5%)	46 (0.8%)	418 (19.1%)
Primary school completed	492 (11.9%)	1479 (16.1%)	390 (7.1%)	484 (22.1%)
Secondary school completed	965 (23.4%)	2322 (25.2%)	3392 (61.4%)	640 (29.2%)
Tertiary education completed	152 (3.7%)	767 (8.3%)	1662 (30.1%)	192 (8.8%)
Marital status				
Never married	141 (3.4%)	348 (3.8%)	303 (5.5%)	354 (14.6%)
Married/cohabiting	2650 (64.3%)	7593 (82.4%)	3231 (58.5%)	1302 (53.6%)
Separated/divorced/widow	1329 (32.3%)	1268 (13.8%)	1986 (36.0%)	772 (31.8%)
Region				
Urban	1561 (37.7%)	2817 (30.6%)	4577 (82.9%)	1810 (72.0%)
Rural	2580 (62.3%)	6393 (69.4%)	946 (17.1%)	704 (28.0%)

The numbers of study participants with and without arthritis according to exposure status for each of the five physical work-related stressors, stratified by country, are presented in Table 2. Overall, the numbers of persons with doctor-diagnosed arthritis was 3778 (17.7%) and with symptom-based arthritis was 1185 (5.9%). Arthritis, using both definitions, was more prevalent among those with physical work-related stressors.

**Table 2:** Country-specific numbers (%) of participants with and without arthritis, across physical work-related stressors

Job exposure, and exposure status		Ghana (*n* = 3924)		India (*n* = 8773)		Russian Federation (*n* = 5144)		South Africa (*n* = 2396)	
		Yes	No	Yes	No	Yes	No	Yes	No
Doctor-diagnosed arthritis									
HPW	Yes	327 (11.1%)	2628 (88.9%)	828 (14.6%)	4822 (85.3%)	470 (34.9%)	875 (65.1%)	205 (18.0%)	931 (81.9%)
	No	118 (9.9%)	1068 (90.0%)	483 (13.6%)	3077 (86.4%)	1132 (27.1%)	3046 (72.9%)	215 (15.6%)	1165 (84.4%)
K/S	Yes	178 (13.9%)	1100 (86.1%)	246 (17.1%)	1194 (82.9%)	210 (34.6%)	396 (65.4%)	178 (22.7%)	605 (77.3%)
	No	267 (9.3%)	2596 (90.7%)	1065 (13.7%)	6705 (86.3%)	1392 (28.3%)	3525 (71.7%)	242 (14.0%)	1491 (86.0%)
HL	Yes	152 (8.6%)	1610 (91.4%)	585 (14.3%)	3492 (85.6%)	187 (34.9%)	349 (65.1%)	36 (10.9%)	293 (89.1%)
	No	293 (12.3%)	2086 (87.7%)	726 (14.1%)	4407 (85.6%)	1415 (28.4%)	3572 (71.6%)	384 (17.6%)	1803 (82.4%)
AE	Yes	5 (3.4%)	143 (96.6%)	53 (24.5%)	163 (75.5%)	138 (39.0%)	216 (61.0%)	9 (9.3%)	88 (90.7%)
	No	440 (11.0%)	3553 (89.0%)	1258 (14.0%)	7736 (86.0%)	1464 (28.3%)	3705 (71.7%)	411 (17.0%)	2008 (83.0%)
AP	Yes	335 (11.1%)	2669 (88.8%)	440 (16.1%)	2292 (83.9%)	228 (32.7%)	470 (67.3%)	64 (20.6%)	246 (79.3%)
	No	110 (9.7%)	1027 (90.3%)	871 (13.4%)	5607 (86.5%)	1374 (28.5%)	3451 (71.5%)	356 (16.1%)	1850 (83.9%)
Symptom-defined arthritis									
HPW	Yes	279 (9.9%)	2538 (90.1%)	191 (3.6%)	5153 (96.4%)	151 (12.0%)	1105 (88.0%)	30 (2.8%)	1057 (97.2%)
	No	134 (12.1%)	973 (87.9%)	41 (1.2%)	3388 (98.8%)	332 (8.5%)	3556 (91.5%)	27 (2.1%)	1282 (97.9%)
K/S	Yes	161 (13.3%)	1046 (86.7%)	55 (4.1%)	1276 (95.9%)	100 (17.2%)	482 (82.8%)	25 (3.4%)	716 (96.6%)
	No	252 (9.3%)	2465 (9.7%)	177 (2.4%)	7265 (97.6%)	383 (8.4%)	4179 (91.6%)	32 (1.9%)	1623 (98.1%)
HL	Yes	128 (7.6%)	1551 (92.4%)	139 (3.6%)	3711 (96.4%)	98 (18.8%)	423 (81.2%)	2 (0.6%)	320 (99.4%)
	No	285 (12.7%)	1960 (87.3%)	93 (1.9%)	4830 (98.1%)	385 (8.3%)	4238 (91.7%)	55 (2.6%)	2019 (97.3%)
AE	Yes	13 (10.7%)	108 (89.3%)	8 (5.1%)	149 (94.9%)	55 (16.2%)	285 (83.8%)	1 (1.1%)	90 (98.9%)
	No	400 (10.5%)	3403 (89.5%)	224 (2.6%)	8392 (97.4%)	428 (8.9%)	4376 (91.1%)	56 (2.4%)	2249 (97.6%)
AP	Yes	273 (9.5%)	2590 (90.5%)	91 (3.5%)	2487 (96.5%)	79 (11.8%)	592 (88.2%)	11 (3.6%)	293 (96.4%)
	No	140 (13.2%)	921 (86.8%)	141 (2.3%)	6054 (97.7%)	404 (9.0%)	4069 (91.0%)	46 (2.2%)	2046 (97.8%)

*Abbreviations*: *HPW* heavy physical work, *K/S* kneeling/squatting, *HL* heavy lifting, *AE* arm elevation, *AP* awkward trunk posture

Results for multivariable associations between physical work-related stressors and doctor-diagnosed or symptom-based arthritis are presented in Tables 3 and 4, respectively. For doctor-diagnosed arthritis, heavy physical work was associated with a 12% increase in the adjusted odds of arthritis (AOR 1.12, 95%CI 1.01–1.23), kneeling or squatting with a 25% increase (AOR 1.25, 95%CI 1.12–1.38), awkward trunk posture with ~ 20% increase (AOR 1.23, 95%CI 1.12–1.36), and arm elevation showed almost a 70% greater odds for arthritis (AOR 1.66, 95%CI 1.37–2.00): no significant association was observed for heavy lifting (*p* = 0.15). For symptom-based arthritis, kneeling or squatting and heavy lifting were each associated with an almost 30% increase in the adjusted odds of arthritis (AOR 1.27, 95%CI 1.08–1.50; AOR 1.33, 95%CI 1.11–1.58, respectively), whilst arm elevation more than doubled the odds of arthritis (AOR 2.16, 95%CI 1.63–2.86); neither heavy physical work nor awkward trunk posture was associated with arthritis (*p* = 0.53; *p* = 0.27, respectively). Factors consistently observed to be significant in the associations between work-related stressors and doctor-diagnosed or symptom-based arthritis were female sex, obesity (BMI ≥30 kg/m^2^), lower income and educational attainment, and being married/cohabiting. Rurality status was only associated with kneeling or squatting, heavy lifting and arm elevation, and only for doctor-diagnosed arthritis.

**Table 3:** Multivariable results (simultaneous adjustments)^a^ for associations between physical work-related stressors and doctor-diagnosed arthritis

Doctor-diagnosed arthritis					
	Model 1	Model 2	Model 3	Model 4	Model 5
	OR (95%CI)	OR (95%CI)	OR (95%CI)	OR (95%CI)	OR (95%CI)
Physical stressors in the Job Exposure Matrix					
Heavy physical work	1.12 (1.01–1.23)*	–	–	–	–
Kneeling/squatting	–	1.25 (1.12–1.38)**	–	–	–
Heavy lifting	–	–	1.08 (0.97–1.20)	–	–
Arm elevation	–	–	–	1.66 (1.37–2.00)**	–
Awkward trunk posture	–	–	–	–	1.23 (1.12–1.36)**
Sex					
Male	1.00	1.00	1.00	1.00	1.00
Female	1.65 (1.51–1.81)**	1.58 (1.44–1.74)**	1.69 (1.54–1.86)**	1.69 (1.54–1.86)**	1.68 (1.54–1.85)**
Age groups (years)					
30–39	1.00	1.00	1.00	1.00	1.00
40–49	3.13 (2.06–4.74)	3.19 (2.10–4.84)	3.11 (2.05–4.72)	3.15 (2.07–4.77)	3.18 (2.10–4.82)
50–59	4.16 (2.74–6.30)	4.21 (2.77–6.38)	4.16 (2.74–6.31)	4.18 (2.75–6.34)	4.22 (2.79–6.41)
60–69	5.73 (3.87–8.46)	5.82 (3.94–8.61)	5.74 (3.89–8.49)	5.87 (3.97–8.68)	5.84 (3.95–8.63)
70–79	8.95 (6.04–13.26)	9.11 (6.15–13.50)	8.97 (6.05–13.28)	9.18 (6.20–13.60)	9.05 (6.11–13.41)
≥80	14.30 (9.63–21.24)	14.54 (9.78–21.59)	14.37 (9.67–21.34)	14.69 (9.89–21.82)	15.57 (9.81–21.65)
Overall *p*-value^b^	**	**	**	**	**
Body mass index (BMI)					
< 25 (kg/m^2^)	1.00	1.00	1.00	1.00	1.00
25–29.99 (kg/m^2^)	1.06 (0.94–1.21)	1.06 (0.93–1.20)	1.07 (0.94–1.21)	1.05 (0.93–1.19)	1.07 (0.94–1.21)
30–34.99 (kg/m^2^)	1.85 (1.59–2.14)	1.83 (1.58–2.12)	1.85 (1.60–2.15)	1.80 (1.55–2.08)	1.85 (1.60–2.14)
≥35 (kg/m^2^)	3.74 (3.19–4.39)	3.70 (3.16–4.34)	3.72 (3.17–4.37)	3.65 (3.11–4.28)	3.76 (3.21–4.41)
Overall *p*-value^b^	**	**	**	**	**
Household income quintiles					
Quintile 1 (lowest)	1.42 (1.24–1.63)	1.46 (1.27–1.68)	1.42 (1.24–1.63)	1.43 (1.24–1.64)	1.45 (1.26–1.66)
Quintile 2	1.38 (1.21–1.58)	1.40 (1.22–1.60)	1.38 (1.21–1.58)	1.37 (1.19–1.57)	1.39 (1.21–1.59)
Quintile 3	1.44 (1.26–1.64)	1.45 (1.27–1.65)	1.44 (1.26–1.64)	1.43 (1.26–1.6)	1.45 (1.27–1.65)
Quintile 4	1.60 (1.42–1.80)	1.61 (1.42–1.81)	1.61 (1.42–1.81)	1.60 (1.42–1.81)	1.60 (1.42–1.80)
Quintile 5	1.00	1.00	1.00	1.00	1.00
Overall *p*-value^b^	**	**	**	**	**
Educational attainment					
No schooling	0.70 (0.59–0.84)	0.70 (0.59–0.83)	0.73 (0.61–0.86)	0.73 (0.61–0.86)	0.70 (0.59–0.83)
Some primary	1.28 (1.06–1.55)	1.28 (1.07–1.55)	1.32 (1.10–1.59)	1.29 (1.07–1.56)	1.28 (1.06–1.54)
Primary complete	0.76 (0.64–0.91)	0.77 (0.65–0.91)	0.79 (0.67–0.93)	0.77 (0.66–0.91)	0.76 (0.65–0.90)
Secondary complete	0.77 (0.67–0.87)	0.77 (0.68–0.87)	0.78 (0.69–0.88)	0.76 (0.67–0.86)	0.76 (0.67–0.86)
Tertiary complete	1.00	1.00	1.00	1.00	1.00
Overall *p*-value^b^	**	**	**	**	**
Marital status					
Never married	1.00	1.00	1.00	1.00	1.00
Married/cohabiting	1.31 (1.03–1.67)	1.31 (1.03–1.66)	1.30 (1.02–1.65)	1.30 (1.02–1.65)	1.28 (1.00–1.62)
Separated/widow	1.16 (0.90–1.48)	1.15 (0.90–1.47)	1.15 (0.90–1.47)	1.15 (0.90–1.48)	1.13 0.88–1.44)
Overall *p*-value^b^	**	**	**	*	*
Region					
Urban	1.00	1.00	1.00	1.00	1.00
Rural	1.09 (0.99–1.20)	1.11 (1.01–1.22)*	1.10 (1.00–1.21)*	1.12 (1.02–1.23)**	1.09 (0.99–1.20)

^a^Analyses were adjusted for country of residence; ^b^ overall *p*-values provided for variables with more than one category; * *p* < 0.05; ** *p* ≤ 0.01

Results presented as adjusted odds ratios (OR) with 95% confidence intervals (95%CI) and *p* value

**Table 4:** Multivariable results (simultaneous adjustments)^a^ for associations between physical work-related stressors and symptom-defined arthritis

Symptom-defined arthritis					
	Model 1	Model 2	Model 3	Model 4	Model 5
	OR (95%CI)	OR (95%CI)	OR (95%CI)	OR (95%CI)	OR (95%CI)
Physical stressors in the Job Exposure Matrix					
Heavy physical work	0.95 (0.81–1.12)	–	–	–	–
Kneeling/squatting	–	1.27 (1.08–1.50)**	–	–	–
Heavy lifting	–	–	1.33 (1.11–1.58)**	–	–
Arm elevation	–	–	–	2.16 (1.63–2.86)**	–
Awkward trunk posture	–	–	–	–	0.91 (0.77–1.07)
Sex					
Male	1.00	1.00	1.00	1.00	1.00
Female	1.44 (1.23–1.69)**	1.33 (1.13–1.57)**	1.62 (1.36–1.94)**	1.50 (1.28–1.75)**	1.43 (1.22–1.67)**
Age groups (years)					
30–39	1.00	1.00	1.00	1.00	1.00
40–49	2.55 (0.97–6.67)	2.65 (1.01–6.94)*	2.53 (0.96–6.61)	2.55 (0.97–6.68)	2.53 (0.96–6.62)
50–59	3.34 (1.28–8.72)	3.36 (1.28–8.78)	3.26 (1.25–8.52)	3.38 (1.29–8.86)	3.31 (1.27–8.66)
60–69	4.35 (1.76–10.74)	4.38 (1.77–10.80)	4.35 (1.76–10.73)	4.43 (1.80–10.94)	4.34 (1.76–10.70)
70–79	10.30 (4.18–25.37)	10.45 (4.24–25.72)	10.44 (4.24–25.69)	10.55 (4.28–26.00)	10.29 (4.18–25.32)
≥80	12.74 (5.16–31.46)	12.65 (5.13–31.24)	12.60 (5.10–31.10)	12.67 (5.13–31.31)	12.70 (5.14–31.33)
Overall *p*-value^b^	**	**	**	**	**
Body mass index (BMI)					
< 25 (kg/m^2^)	1.00	1.00	1.00	1.00	1.00
25–29.99 (kg/m^2^)	1.14 (0.92–1.40)	1.15 (0.93–1.41)	1.16 (0.94–1.42)	1.13 (0.92–1.39)	1.13 (0.92–1.40)
30–34.99 (kg/m^2^)	1.35 (1.04–1.75)	1.38 (1.06–1.79)	1.39 (1.07–1.81)	1.33 (1.02–1.72)	1.34 (1.03–1.74)
≥35 (kg/m^2^)	3.49 (2.68–4.56)	3.62 (2.78–4.72)	3.65 (2.80–4.76)	3.47 (2.67–4.52)	3.47 (2.66–4.52)
Overall *p*-value^b^	**	**	**	**	**
Household income quintiles					
Quintile 1 (lowest)	1.00 (0.80–1.25)	1.03 (0.82–1.29)	0.98 (0.77–1.21)	0.99 (0.79–1.24)	1.00 (0.80–1.25)
Quintile 2	1.24 (1.00–1.54)	1.25 (1.01–1.55)	1.22 (0.98–1.50)	1.19 (0.96–1.48)	1.24 (1.00–1.54)
Quintile 3	0.62 (0.49–0.79)	0.63 (0.50–0.80)	0.61 (0.48–0.77)	0.61 (0.48–0.78)	0.62 (0.49–0.79)
Quintile 4	0.68 (0.54–0.86)	0.69 (0.55–0.86)	0.67 (0.53–0.84)	0.68 (0.54–0.85)	0.68 (0.54–0.86)
Quintile 5	1.00	1.00	1.00	1.00	1.00
Overall *p*-value^b^	**	**	**	**	**
Educational attainment					
No schooling	7.15 (4.84–10.57)	6.54 (4.47–9.56)	6.28 (4.28–9.20)	6.71 (4.60–9.79)	7.13 (4.87–10.44)
Some primary	9.76 (6.55–14.53)	9.13 (6.18–13.51)	8.83 (5.96–13.09)	9.11 (6.17–13.47)	9.69 (6.55–14.34)
Primary complete	3.02 (2.04–4.48)	2.84 (1.93–4.18)	2.78 (1.89–4.10)	2.78 (1.89–4.09)	3.01 (2.04–4.44)
Secondary complete	2.83 (2.09–3.84)	2.72 (2.01–3.69)	2.71 (2.00–3.68)	2.64 (1.95–3.58)	2.84 (2.09–3.85)
Tertiary complete	1.00	1.00	1.00	1.00	1.00
Overall *p*-value^b^	**	**	**	**	**
Marital status					
Never married	1.00	1.00	1.00	1.00	1.00
Married/cohabiting	1.05 (0.67–1.63)	1.04 (0.66–1.62)	1.01 (0.65–1.58)	1.04 (0.67–1.62)	1.06 (0.68–1.66)
Separated/widow	1.22 (0.78–1.91)	1.19 (0.76–1.86)	1.20 (0.76–1.87)	1.23 (0.79–1.93)	1.23 (0.79–1.93)
Overall *p*-value^b^	NS	NS	NS	NS	NS
Region					
Urban	1.00	1.00	1.00	1.00	1.00
Rural	1.11 (0.95–1.30)	1.08 (0.92–1.27)	1.07 (0.91–1.25)	1.09 (0.93–1.28)	1.11 (0.95–1.30)

^a^Analyses were adjusted for country of residence; ^b^ overall *p*-values provided for variables with more than one category; * *p* < 0.05; ** *p* ≤ 0.01; NS = not significant

Results presented as adjusted odds ratios (OR) with 95% confidence intervals (95%CI) and *p* value

Two-way interaction terms between sociodemographic characteristics and work-related stressors for doctor-diagnosed (lifetime) arthritis or symptom-based (current) arthritis are presented in Table 5. The major findings for interaction terms related to social determinants and doctor-diagnosed arthritis are as follows. For the four stressors of heavy physical work, kneeling or squatting, heavy lifting, and awkward trunk posture, every one level increase in quintile of household income (higher income) showed > 10% additional increased odds for doctor-diagnosed arthritis. For heavy lifting, every one increase in the level of educational attainment increased the odds by 10%, whilst for arm elevation the odds of doctor-diagnosed arthritis was decreased by 20%. For symptom-based (current) arthritis, and for all physical stressors, every one level increase in category of educational attainment additionally increased the odds by between 40 and 55% (range: awkward trunk posture, to heavy physical work, respectively). Varying interaction terms were observed for BMI, depending on the definition of arthritis and physical stressor. No country-specific interaction terms were identified, indicating that no further country-specific models were required.

**Table 5:** Two-way interaction terms for associations between physical work-related stressors and arthritis

	Heavy physical work		Kneeling or squatting		Heavy lifting		Arm elevation		Awkward trunk posture	
	OR (95%CI)	*p*	OR (95%CI)	*p*	OR (95%CI)	*p*	OR (95%CI)	*p*	OR (95%CI)	*p*
Doctor-diagnosed arthritis										
Age (years)^a^	0.96 (0.90–1.03)	0.27	1.06 (0.97–1.51)	0.20	0.93 (0.86–1.00)	**0.04**	1.02 (0.87–1.19)	0.81	0.99 (0.92–1.07)	0.85
Body mass index^a^	0.88 (0.81–0.96)	**0.006**	1.01 (0.91–1.13)	0.80	0.96 (0.87–1.06)	0.46	1.43 (1.12–1.82)	**0.004**	0.76 (0.69–0.84)	**≤0.001**
Household income^a^	1.13 (1.06–1.20)	**≤0.001**	1.12 (1.04–1.20)	**0.003**	1.10 (1.03–1.18)	**0.005**	0.99 (0.85–0.15)	0.90	1.08 (1.01–1.15)	**0.03**
Education^a^	1.03 (0.97–1.11)	0.32	1.00 (0.92–1.08)	0.97	1.09 (1.01–1.17)	**0.02**	0.80 (0.66–0.96)	**0.02**	1.03 (0.96–1.10)	0.42
Never married	1.23 (0.76–1.99)	0.40	1.51 (0.89–2.57)	0.13	0.84 (0.43–1.67)	0.62	0.52 (0.13–2.04)	0.35	0.60 (0.25–1.44)	0.25
Married/cohabiting	1.22 (1.02–1.47)	**0.03**	1.17 (0.94–1.46)	0.16	1.12 (0.90–1.39)	0.32	0.95 (0.59–0.53)	0.83	1.40 (1.15–1.71)	**0.001**
Separated/widow^b^	1.00	–	1.00	–	1.00	–	1.00	–	1.00	–
Symptom-defined arthritis										
Age (years) ^a^	0.94 (0.82–1.07)	0.35	1.47 (1.26–1.71)	**≤0.001**	1.02 (0.89–1.16)	0.80	1.83 (1.27–2.63)	**0.001**	1.12 (0.97–1.28)	0.11
Body mass index^a^	0.58 (0.49–0.68)	**≤0.001**	0.97 (0.81–1.15)	0.70	0.83 (0.71–0.98)	**0.03**	1.53 (1.03–2.30)	**0.04**	0.69 (0.59–0.81)	**≤0.001**
Household income^a^	1.10 (1.00–1.22)	0.06	1.10 (0.99–1.23)	0.08	1.03 (0.92–1.15)	0.53	1.05 (0.83–1.32)	0.70	1.11 (1.00–1.23)	**0.048**
Education^a^	1.55 (1.39–1.73)	**≤0.001**	1.45 (1.28–1.65)	**≤0.001**	1.49 (1.33–1.68)	**≤0.001**	1.45 (1.03–2.03)	**0.03**	1.44 (1.29–1.61)	**≤0.001**
Never married	0.89 (0.37–2.19)	0.81	1.53 (0.53–4.48)	0.43	0.29 (0.06–1.31)	0.11	^c^	^c^	0.19 (0.02–1.47)	0.11
Married/cohabiting	1.16 (0.87–1.55)	0.32	2.92 (2.10–4.04)	**≤0.001**	0.87 (0.63–1.21)	0.42	3.12 (1.53–6.33)	**0.002**	1.12 (0.84–1.50)	0.44
Separated/widow^b^	1.00	–	1.00	–	1.00	–	1.00	–	1.00	–

^a^Variables treated as ordinal in models; ^b^reference group; ^c^empty cells due to small counts; significant interaction terms are bolded

## Discussion

In our study population from the LMICs of Ghana, India, Russian Federation and South Africa, four of the five work-related stressors were associated with increased odds of doctor-diagnosed arthritis (heavy physical work, kneeling or squatting, arm elevation, and awkward trunk posture), while three stressors increased the odds of symptom-defined arthritis (kneeling or squatting, heavy lifting, and arm elevation). Obesity, defined as a BMI of ≥30 kg/m^2^, was independently associated with doctor-diagnosed (lifetime) and symptom-based (current) arthritis, as were the social determinants of lower income and lower educational attainment. Being married/cohabiting was associated with doctor-diagnosed, but not symptom-based, arthritis. We observed that excess risk for arthritis, due to statistical two-way interactions, may exist for those with higher BMI, and that those with higher income are more likely to be diagnosed with arthritis, whilst those with higher education are more likely to report symptoms compared to those with lower education.

Musculoskeletal pain associated with arthritis poses a significant threat to exacerbating poverty in LMICs, as physical ability can be imperative to livelihoods and associations between arthritis and occupation has potential to worsen poverty for those affected. Common work-related exposures in LMICs include sustained squatting or kneeling over longer periods of time, carrying heavy loads for long distances, and climbing up and down steep terrain [25, 26]: occupations that are related to agricultural or cleaning tasks, or transporting food, water and/or building supplies, amongst others. Our findings show that heavy physical work was the most common physical job stressor in each LMIC, and that heavy lifting and kneeling/squatting were associated with increased odds of arthritis in our study population. Importantly, our study includes older persons from LMICs, and as such there is potential for a healthy worker selection; the implications of which are an underestimation of associations between occupational exposures and arthritis. Our data suggests that attention be directed toward addressing work-related kneeling or squatting and arm elevation (repetitive arm lifting movements, and/or sustained arm elevation) in LMICs to reduce arthritis burden. Minimization of occupational risk factors has already been underway for many years in high income countries; indeed, countries such as Denmark and Germany have acknowledged that knee osteoarthritis is an occupational disease [27]. Compounding this issue in LMICs, and despite advances in diagnosis and treatment of arthritis during the last few decades in higher income countries [28], these advances have not impacted on LMICs, which are primarily resource-poor. In addition, there is a wide variation in occupational structures, conditions of work, quality of the work environment, and health status of workers in different regions of the world, and different sectors of economies [29]. In LMICs, there is a greater prevalence of small-scale industrial and agricultural enterprises, which are characterised by fewer resources, heavier workloads, and often necessitates longer hours working and therefore increased exposure to stressors [29]. Multifactorial interventions would be necessary, including screening for pre-existing musculoskeletal conditions, ergonomic modifications, adequate medical treatment, and, in the first instance, equitable access to healthcare for diagnosis and disease management. Indeed, greater access to formal education would increase the ability of populations from LMICs to access healthcare.

We speculate as to why some of the physical work-related stressors were associated with doctor-diagnosed arthritis but not symptom-related arthritis, and vice versa. These differences may be related to the time element that is inherent in the diagnosis of arthritis which encompasses the longer-term of ‘ever’, whilst symptom-based refers to the more ‘current’ period of time. For instance, having already been diagnosed with arthritis is more likely to mean that symptoms have been present over a longer-term, and that some systemic damage to the skeletal system may be present. Self-reported doctor diagnosis of arthritis is more likely to identify inflammatory arthritis as well as osteoarthritis, thereby resulting in different observations in terms of work-related stressors. Individuals who answered ‘yes’ to the symptomatic arthritis question but not the diagnosis question may have less severe arthritis, in that they may not have sought medical care and thus received a diagnosis. In addition, long-term arthritis status may explain why four of the five work related stressors were associated with arthritis, given the potential of movements to aggravate an existing condition where joint damage may already be present, as opposed to only three of the stressors observed to be associated with current symptoms of a condition that may not be diagnosed and thus untreated. Notably, the three stressors associated with symptom-related arthritis were kneeling/squatting, heavy lifting, and arm elevation, most of which have been previously reported as being associated with arthritis [2]. Furthermore, the prevalence of other non-communicable diseases in SAGE varies markedly when defined by self-reported diagnosis compared to standardized criteria, suggesting that, amongst the poorest of the poor, there is more likely to be under-diagnosis and under-reporting of non-communicable diseases [30, 31]. These differences may also plausibly be influenced by variations between countries in health literacy, access to care, and how health is understood.

LMICs are no longer absent from discussions regarding the increasing prevalence of obesity; indeed, we observed that more than 70% of our study populations from Russian Federation and South Africa were in Classes 1 and 2 of the obese BMI categories. We acknowledge, however, that some of that proportion may plausibly be related to the greater weight, muscle or bone mass of these populations, rather than excess body fat. Whilst our study populations from Ghana and Indian were primarily in the normal BMI category, almost 25% of those from Ghana and 13% of those from India were represented in the overweight and obese BMI categories. A study that focused on the Ghana population from SAGE showed that BMI in the obese category increased the likelihood of arthritis (OR 1.65, 95%CI 1.14–2.39) [32]. Our data suggest that BMI of ≥30 kg/m^2^ is independently associated with arthritis in all four countries investigated; however, and except for arm elevation, BMI did not explain the associations between work-related stressors and the likelihood of arthritis. The implications of these data are that weight management may be especially critical for workers that are exposed to repeated arm elevation movements, and indeed weight management should form an important component of primary and secondary arthritis prevention in LMICs, as for higher income countries.

Our investigations into statistical two-way interaction terms highlighted some interesting findings regarding associations between social determinants and arthritis. For each of the work-related stressors (with the exception of arm elevation), significant interaction terms suggested that individuals with higher income were more likely to report a doctor-diagnosis of arthritis. For many individuals and households in LMICs, there are inadequate financial resources to manage the cost of a chronic disease such as arthritis, with an impoverishing effect of paying for healthcare services out-of-pocket [33]. These significant interaction terms may be indicative of the increased likelihood for individuals with higher income to have the resources to access a doctor for a diagnosis. Indeed, a study of 4724 adults aged ≥50 years from Ghana [32] also reported that the prevalence of doctor-diagnosed arthritis was greater for those with the highest compared to the lowest income (16.1% vs 11.0%). Another issue that may influence a doctor-diagnosis is the geographical location: the availability of doctors is metro-centric, and likely related to the overall socioeconomic status of communities. For symptom-defined arthritis, and for each of the work-related stressors, consistently significant interaction terms suggested that individuals with higher education were more likely to have symptom-based arthritis. We speculate that higher educated persons may have higher expectations of their physical health, and/or are more apt to verbalise symptoms compared to less educated persons who may ‘accept their lot’. Indeed, cultural variations in the way that pain is perceived, and linguistic variation in the way that pain is defined and classified, have been identified [25, 34, 35].

It was promising that no country-specific interaction terms were identified; a result likely indicating that (i) our ‘pooled across countries’ models that adjusted for country may explain associations between exposures and risk factors and (lifetime) arthritis, and (ii) during the development of the JEM [21, 22], any potential between-country differences in physical work-related stressors were addressed. Although the JEM was developed using Finnish population-based data, that we did not observe any country-specific two-way interactions between job stressors and arthritis also suggests that the use of the ISCO-88 introduces comparability and thus increases the potential for use of the JEM in other countries. Although the ISCO-88 is now succeeded by the ISCO-08, the International Labour Organization reported in 2010 that the principles and top structure of ISCO-88 correspond with the more recent ISCO-08 version [36].

Our study has several strengths. The JEM is valid for physical exposure assessment in large-scale epidemiological assessments [21, 22], and it has been reported to have relatively high specificity, with little compromise on sensitivity [21]. The SAGE study consists of a large, multi-national cohort, with nationally representative samples. Our sample size provided sufficient statistical power to detect even small effect sizes on interactions. SAGE data were collected using standardized survey instruments and methodologies thus ensuring consistency. The integrity of SAGE data is overseen by WHO, collaborating closely with leading institutions from each of the countries involved and with national health authorities [16]. Ours is the first study to link data from populations of LMICs to a JEM to investigate the role of physical work-related stressors on arthritis risk. The method of exposure assessment employed for the development of the JEM is less likely to be prone to recall bias and confers a degree of objectivity. The use of the JEM also limits the likelihood of recall bias regarding work-related exposure. Our study also has some limitations. Although the JEM was initially developed to identify high-risk occupations for lower back pain, the mechanical physical stressors included within the JEM are also appropriate for the investigation of osteoarthritis. However, we are unable to identify which joints were affected by arthritis. There is a possibility that non-differential misclassification of work-related stressors may lead to the attenuation of the observed associations toward null [21]. However, should there be greater physical work-related stressors in LMICs compared to Finland, for instance due to variation in work processes associated with specific jobs, this would result in our estimates being conservative and thus an underestimation of associations. Cumulative years in specific jobs would likely increase the risk of arthritis, as has been shown by others [37], thus whilst cumulative data was not available, this would also result in an underestimation of associations. Data were not available regarding specific types of arthritis diseases; however, we investigated the symptom-based algorithm in order to identify symptoms most likely associated with osteoarthritis rather than inflammatory arthritis. This was important, as the physical stressors measured by the JEM are also more likely to be associated with osteoarthritis. Due to the nature of the data, we cannot analyse the time pressure and safety-related issues in each work place, not for each individual country or person. Such stressors in the workplace have been shown to biopsychosocial impacts which can alter pain modulation and sensation.

## Conclusions

In conclusion, these data suggest that attention be directed toward addressing work-related kneeling/squatting and arm elevation in LMIC to reduce arthritis burden, especially given that minimization of occupational risk factors has been underway for many years in high income countries. Observed excess risk for arthritis, due to interactions, appears to exist for those with BMI in the overweight and obese categories. In addition, individuals with higher income are more likely to be diagnosed with arthritis, whilst those with higher education are more likely to report symptoms. Future studies could evaluate whether (i) there is a threshold time of exposure to physical work-related stressors to the development of arthritis, and (ii) arthritis is a determinant of reduced work capacity in those exposed to these physical risk factors in their employment.

## Additional file

Additional file 1: **Table S1:** Symptom-based questions and related algorithm to ascertain prevalent arthritis. (DOCX 13 kb)

## Abbreviations

BMI: Body mass index
CI: Confidence intervals
ISCO-88: International Standard Classification of Occupations (1988)
JEM: Job Exposure Matrix
LMICs: Lower- and middle-income countries
OR: Odds ratios
SAGE: Study on global AGEing and adult health
WHO: World Health Organization

## References

[1] McMillan G, Nichols L (2005). Osteoarthritis and meniscus disorders of the knee as occupational diseases of miners. Occup Environ Med.

[2] Jensen LK (2008). Knee osteoarthritis: influence of work involving heavy lifting, kneeling, climbing stairs or ladders, or kneeling/squatting combined with heavy lifting. Occup Environ Med.

[3] Palmer KT (2012). Occupational activities and osteoarthritis of the knee. Brit Med J.

[4] Teichtahl AJ, Smith S, Wang Y, Wluka AE, O'Sullivan R, Giles GG (2015). Occupational risk factors for hip osteoarthritis are associated with early hip structural abnormalities: a 3.0 T magnetic resonance imaging study of community-based adults. Arthrit Res Ther.

[5] Sulsky SI, Carlton L, Bochmann F, Ellegast R, Glitsch U, Hartmann B (2017). Epidemiological evidence for work load as a risk factor for osteoarthritis of the hip: a systematic review. PLoS One.

[6] Lievense A, Bierma-Zeinstra SMA, Verhagen AP, Verhaar JAN, Koes BW (2001). Influence of work on the development of osteoarthritis of the hip: a systematic review. J Rheumatol.

[7] Olsson AR, Skogh T, Axelson O, Wingren G (2004). Occupations and exposures in the work environment as determinants for rheumatoid arthritis. Occup Environ Med.

[8] Kirkhorn S, Greenlee RT, Reeser JC (2003). The epidemiology of agriculture-related osteoarthritis and its impact on occupational disability. Wisconsin Med J.

[9] Murrell SA, Meeks S (2002). Psychological, economic, and social mediators of the education-health relationship in older adults. J Aging Health.

[10] Meeks S, Murrell SA (2001). Contribution of education to health and life satisfaction in older adults mediated by negative affect. J Aging Health.

[11] White CM, PD SJ, Cheverie MR, Iraniparast M, Tyas SL (2015). The role of income and occupation in the association of education with healthy aging: results from a population-based, prospective cohort study. BMC Public Health.

[12] Brennan-Olsen SL, Cook S, Leech MT, Bowe SJ, Kowal P, Naidoo N, Ackerman IN, Page RS, Hosking SM, Pasco JA, Mohebbi M (2017). Prevalence of arthritis according to age, sex and socioeconomic position in six low and middle income countries: analysis of data from the World Health Organization study on global AGEing and adult health (SAGE) wave 1. Ann Rheum Dis.

[13] Loring B, Robertson A, WHO (2014). Obesity and inequities: Guidance for addressing inequities in overweight and obesity.

[14] WHO. Obesity: Preventing and managing the global epidemic. Report of a WHO Consultation. WHO Technical Report Serues 894. Geneva: WHO, 2000. 11234459

[15] Cicuttini FM, Wluka AE (2016). Not just loading and age: the dynamics of osteoarthritis, obestiy and inflammation. Med J Aust.

[16] Kowal P, Chatterji S, Naidoo N, Biritwum R, Fan W, Ridaura RL, Maximova T, Arokiasamy P, Phaswana-Mafuya N, Williams S, Snodgrass JJ, Minicuci N, E'Este C, Peltzer K, Boerma JT (2012). The SAGE collaborators. Data resource profile: the World Health Organization study on global AGEing and adult health (SAGE). Int J Epidemiol.

[17] Chatterji S (2013). World Health Organization's (WHO) study global AGEing and adult health (SAGE). BMC Proc.

[18] STROBE Statement: Strengthening the reporting of observational studies in epidemiology. University of Bern. 2007. 10.1136/bmj.39335.541782.AD PMC2034723 17947786

[19] ILO (1990). International Standard Classification of Occupations (ISCO-88).

[20] ILO. International statistical comparisons of occupational and social structures: problems, possibilities and the role of ISCO-88, vol. 2004. Geneva: International Labour Organization.

[21] Solovieva S, Pehkonen I, Pensola T, Haukka E, Kausto J, Leivategija T, Shiri R, Heliovaara M, Burdorf A, Husgafvel-Pursiainen K, Viikari-Juntura E. Development of physical and psychosocial job exposure matrices. Helsinki: Finnish institute of. Occupational Health. 2014;

[22] Solovieva S, Pehkonen I, Kausto J, Miranda H, Shiri R, Kauppinen T, Heliovaara M, Burdorf A, Husgafvel-Pursiainen K, Viikari-Juntura E (2012). Development and validation of a job exposure matrix for physical risk factors in low back pain. PLoS One.

[23] WHO (2004). Appropriate body mass index for Asian populations and its implications for policy and intervention strategies. Lancet.

[24] UNESCO. International Standard Classification of Education: ISCED 1997, 2006. http://uis.unesco.org/sites/default/files/documents/international-standard-classification-of-education-isced-2011-en.pdf.

[25] Hoy DG, Fransen M, March L, Brooks P, Durham J, Toole MJ (2010). In rural Tibet, the prevalence oflower limb pain, especially knee pain, is high: an observational study. J Physiotherapy.

[26] Cozzensa da Silva M, Fassa AG, Rodrigues Domingues M, Kriebel D (2007). Knee pain and associated occupational factors: a systematic review (translated). Cademos de Saude Publica.

[27] Ezzat AM, Li LC (2014). Occupational physical loading tasks and knee osteoarthritis: a review of the evidence. Physiother Can.

[28] Storheim K, Zwart J-A (2014). Musculoskeletal disorders and the global burden of disease study. Ann Rheum Dis.

[29] WHO (1994). Global strategy on occupational health for all: The way to health at work. Recommendation of the second meeting of the WHO Collaborating Centres in Occupational Health. Trends of global economies.

[30] Vellakkal S, Subramanian SV, Millett C, Basu S, Stuckler D, Ebrahim S (2013). Socioeconomic inequalities in non-communicable diseases prevalence in India: disparities between self-reported diagnoses and standardized measures. PLoS One.

[31] Vellakkal S, Millett C, Basu S, Khan Z, Aitsi-Selmi A, Stuckler D, Ebrahim S (2015). Are estimates of socioeconomic inequalities in chronic disease artefactually narrowed by self-reported measures of prevalence in low-income and middle-income countries? Findings from the WHO-SAGE survey. J Epidemiol Comm Health.

[32] Minicuci N, Biritwum RB, Mensah G, Yawson AE, Naidoo N, Chatterji S, Kowal P (2014). Sociodemographic and socioeconomic patterns of chronic non-communicable disease among the older population in Ghana. Glob Health Action.

[33] Xu K, Evans DB, Kawabata K, Zeramdini R, Klavus J, Murray CJ (2003). Household catastrophic health expenditure: a multicountry analysis. Lancet.

[34] David M, Braun T, Borde T (2004). Pain and ethnicity-results of a survey at three internal/gynecological first-aid stations in Berlin (translated). Zentralblatt fur Gynakologie.

[35] Gureje O, Von Korff M, Simon GE, Gater R (1998). Persistent pain and well-being: a World Health Organization study in primary care. JAMA.

[36] ILO. ISCO-08 Structure, index correspondence with ISCO-88. Geneva: International Labour Organization, 2016.

[37] Andersen S, Caspar-Thygesen L, Davidsen M, Helweg-Larsen K (2012). Cumulative years in occupation and the risk of hip or knee osteoarthritis in men and women: a register-based follow-up study. Occup Environ Med.

