# Supplementary material for: Arthritis diagnosis and symptoms are positively associated with specific physical job exposures in lower- and middle-income countries: cross-sectional results from the World Health Organization’s Study on global AGEing and adult health (SAGE)
Source: BMC Public Health. 2018 Jun 8;18:719. doi: 10.1186/s12889-018-5631-2 (PMC5994040; doi:10.1186/s12889-018-5631-2)
Supplement: Supplementary file 1 — Table S1: Symptom-based questions and related algorithm to ascertain prevalent arthritis. (DOCX 13 kb) [file 12889_2018_5631_MOESM1_ESM.docx]

**Additional file 1: Table S1:** Symptom-based questions and related algorithm to ascertain prevalent arthritis

| **Question number** | **Question text and algorithm** |
| --- | --- |
| 1 | During the last 12 months, have you experienced pain, aching, stiffness or swelling in or around the joints (like arms, hands, legs or feet) which were not related to an injury and lasted for more than a month? |
| 2 | During the last 12 months, have your experienced stiffness in the joint in the morning after getting up from bed, or after a long rest of the joint without movement? |
| 3 | Did this stiffness last for more than 30 minutes? |
| 4 | Did this stiffness go away after exercise or movement in the joint? |
| *Algorithm* | *If a participant responded with ‘yes’ to questions 1 and/or 2, and responded with ‘no’ to questions 3 and 4, then the participant was categorised as most likely having osteoarthritis (rather than inflammatory arthritis)* |
